# Risk factors for drug-treated major adverse cardio-cerebrovascular events in patients on primary preventive statin therapy: A retrospective cohort study

**DOI:** 10.1016/j.pmedr.2023.102258

**Published:** 2023-05-29

**Authors:** Dennis Steenhuis, Stijn de Vos, Jens H.J. Bos, Eelko Hak

**Affiliations:** University of Groningen, Groningen Research Institute of Pharmacy, Unit of PharmacoTherapy, -Epidemiology & -Economics, A. Deusinglaan 1, 9713 AV Groningen, the Netherlands

**Keywords:** Statin therapy, HMG-CoA reductase inhibitors, Primary prevention, Cardio-cerebrovascular events, Pharmacoepidemiology, Risk factors, weighted time-dependent Cox proportional hazard model

## Abstract

We aim to identify risk factors of major adverse cardio-cerebrovascular events (MACCE) using a proxy of drug treatment for a MACCE after the start of statin therapy in the primary cardiovascular prevention group, taking drug dose, persistency and adherence into account. We conducted a retrospective inception cohort study using data from the University of Groningen prescription database IADB.nl, covering patients in the Northern part of the Netherlands. We identified adult starters on primary preventive statin therapy as patients without any statin or cardiovascular drug prescription in the two years before the first statin dispensing and used a weighted Cox proportional hazard model to estimate hazard ratios (HR) with their 95 % confidence intervals (95 %CI). Among 39,487 primary preventive statin starters, 23% received drug treatment for a MACCE within a median follow-up period of four years. Increasing age, male gender and presence of diabetes drug treatment were significantly associated with the outcome (HR: 1.03; 95 %CI: 1.02–1.04; HR: 1.27; 95 %CI: 1.12–1.44 and HR: 1.39; 95 %CI: 1.24–1.56, respectively). If patients remained statin therapy persistent, adherence was no longer associated with drug treatment for a MACCE. In 23 % of the statin therapy initiators, incident drug treatment for a MACCE occurred with a median of four years. To reduce event rates in this group, older patients, males and diabetes patients should be closely monitored. Non-adherence in the early stage of treatment should be avoided to prevent non-persistence.

## Highlights

• Long-term risks of MACCE in primary preventive statin therapy.
• Using statin type, dose, adherence and diabetes as time-dependent variables.
• Incident drug-treated MACCE is still very common in primary preventive patients.
• Adherence is no longer associated with drug-treated MACCE in statin-persistent patients.

## 1 Introduction

In 2020, almost two million Dutch inhabitants were using 3-hydroxy-3-methylglutaryl-coenzyme A (HMG-CoA) reductase inhibitors or statins (GIPdatabank, 2021, WHOCC, 2019). Statin therapy reduces the risk of any major cardio- and cerebrovascular events in both primary and secondary prevention, as evidenced by large randomized controlled trials (Cholesterol Treatment Trialists Collaboration, 2010). In the Netherlands, primary cardiovascular prevention with statin therapy is initiated if the predicted risk of experiencing a cardio-cerebrovascular event within the next ten years is more than 5 %, and/or any of the following diseases is present: a previous cardio-cerebrovascular event, diabetes type II or chronic kidney failure. The Dutch college of General Practitioners (GP) agree with guidelines in most other Western countries (Nederlandse Huisartsen Genootschap, 2019).

Statins are used to reduce Low-Density Lipoprotein Cholesterol (LDL-C) levels. Depending on the effective dose, statins can reduce these LDL-C levels by up to 60 % (Feingold, 2000). With the reduction of one mmol/L LDL-C after one year, the risk of developing any major cardiovascular event is reduced by 22 % (Cholesterol Treatment Trialists Collaboration, 2010). Furthermore the risk of developing a fatal or non-fatal stroke, cardiovascular disease or cerebrovascular heart disease is reduced by 38 % (hazard ratio [HR]: 0.62, 95 % confidence interval [95 %CI]: 0.58 to 0.73) by using statins if compared to placebo/usual care, according to a *meta*-analysis by Taylor et al. (2013).

In an exploratory study from our group using a small sample of patients (N = 602), we showed that modifiable risk factors of cardiovascular events at initiation of statin therapy such as LDL-C levels and blood pressure, did no longer play a role in the risk of cardiovascular events, if patients were persistent with this therapy (Steenhuis et al., 2022). This was in line with earlier research done by Corrao et al., 2010, Rannanheimo et al., 2015, who estimated the effect of statin adherence on cardiovascular events in patients on primary preventive statin therapy, adjusted for time-independent risk factors as age, sex, drug dose and diabetes at baseline. Since drug adherence can change over time and a larger sample is needed to confirm our earlier findings, we used the widely researched and large University of Groningen IADB.nl prescription database to examine the role of traditional risk factors of premature cardiovascular drug treatment in primary prevention taking persistence, adherence, statin type and dosing of statin therapy into account.

## 2 Methods

### 2.1 Study design, setting and data source

We conducted a retrospective population-based inception cohort study using the widely researched prescription database IADB.nl of the University of Groningen as a source database. This database contains prescription data from approximately 100 pharmacies for more than 20 years. It covers about 1,200,000 patients in the Northern part of the Netherlands. Registration in the database is independent of health insurance. Age, gender and prescription rates in the database are representative for the Netherlands as a whole. Since the IADB.nl makes use of an unique anonymous identifier, patient anonymity is guaranteed, hence the ethical approval for studies using the IABD.nl database has been waived (Visser et al., 2013).

### 2.2 Study population

The study population consists of adult patients 18 years or older, registered at an IADB.nl pharmacy, who started statins for primary prevention of cardiovascular events anywhere between 1996 and 2019.

### 2.3 Inclusion and exclusion criteria

We selected patients in the IADB.nl database who were 18 years or older at the index date (defined as their first statin prescription). Patients should have received at least two statin prescriptions within a year of their first statin prescription and had at least two years of history in the IADB.nl database before they started statin treatment.

To exclude patients with a history of cardio- or cerebrovascular events at the index date, we excluded patients that had a prescription of any of the following medications in the two years before or within 90 days after the index date: Vitamin K antagonists with the Anatomical Therapeutic Classification (ATC)-code B01AA, Platelet aggregation inhibitors (B01AC), Organic Nitrates (C01DA) or Other vasodilators used in cardiac diseases (C01DX) (WHOCC, 2020a, WHOCC, 2007) used in the acute treatment of cardiovascular events. These drug classes were selected based on validation study of Pouwels et al. (2016), who examined the validity of applying these proxy medications for the exclusion of patients with a history of cardiovascular disease using a pharmacy-dispensing database. Combined, history of a major adverse cardio-cerebrovascular event (MACCE) diagnosed in either a hospital or by a GP is identified by these four medications with a sensitivity of 85 % and a specificity of 75 %.

Patients that started statin therapy with a statin in the “High” Equalized Statin Dose (ESD) class (see Table 1 and subsection 2.5) were excluded since statins in these dosage are mainly prescribed to patients for secondary prevention of cardio-cerebrovascular events (Visseren et al., 2021).

**Table 1:** Equalized statins doses. Doses of statins are combined such that they have approximately the same effect on lowering LDL-C.

ESD	Low		Medium			High		
Atorvastatin			10 mg	20 mg		40 mg	80 mg	
Atorvastatin + 10 mg Ezetimibe			5 mg			10 mg	20 mg	40 mg
Cerivastatin	0.1 mg	0.2 mg	0.4 mg	0.8 mg				
Fluvastatin	20 mg	40 mg	80 mg					
Lovastatin	10 mg	20 mg	40 mg	80 mg				
Pitavastatin			1 mg	2 mg		4 mg		
Pravastatin	10 mg	20 mg	40 mg	80 mg				
Simvastatin	10 mg		20 mg	40 mg	60 mg	80 mg		
Simvastatin + 10 mg Ezetimibe			10 mg			20 mg	40 mg	80 mg
Rosuvastatin			5 mg			10 mg	20 mg	40 mg

**Notes**: Low lowers the LDL-C 0–30%, Medium lowers the LDL-C 30–45% and High more than 45% (Adams et al., 2020, Davidson et al., 2002, Feingold, 2000, Foody et al., 2014).

### 2.4 Outcome

The outcome is drug treatment for major adverse cardio-cerebrovascular events as a proxy for any major adverse cardio-cerebrovascular event on the basis of at least one prescription of the following drugs, Vitamin K antagonists with ATC-code B01AA, Platelet aggregation inhibitors (B01AC), Organic nitrates (C01DA) or Other vasodilators used in cardiac diseases (C01DX) (WHOCC, 2020a, WHOCC, 2007) after statin initiation (Pouwels et al., 2016). Using these four proxy medications, we cover about 57 % of the patients with an encoded incident GP or hospital diagnoses of cerebrovascular event or major ischemic heart disease (IHD) (Pouwels et al., 2016). Pouwels et al. (2016) showed that only 6 % of the patients without these acute cardiovascular medications should have classified as a patient with a cardio-cerebrovascular event. We defined the time-to-event as the time from the index-date (first statin prescription) to the first prescription of any of these drug-treatments, whichever came first. Patients were censored when they did not receive one of the abovementioned medications, when they reached the end of the study or left the IADB.nl database.

### 2.5 Covariates

Information on age and gender was collected at the index date. We grouped combinations of statins and dose together, such that these lower the LDL-C with about the same percentage points (Adams et al., 2020, Davidson et al., 2002, Feingold, 2000, Foody et al., 2014). Grouping occurred into three so-called ESD-classes (Equalized Statin Dose): Low: less than 30 % LDL-C reduction; Medium: 30–45 % LDL-C reduction; and High: more than 45 % LDL-C reduction; (Table 1). Note that the ESD class is a direct derivative of the statin type. Using a standard permissible gap model, we defined patients as having discontinued statin medication and therefore being non-persistent when the prescription gap exceeds three times the dispensed daily supply of the last prescription (Andrade et al., 2006). By combing the amount of dispensed medication and the dispensing dates, we measured the adherence to statin therapy using the Continuous, Multiple Interval Measure of Medication Acquisition (CMA) (Lam and Fresco, 2015). We categorized the statin adherence into two classes, 1: High adherence, more than 80 %; and 2. Low adherence, 0–80 %. Note that by definition non-persistent patients have an average adherence less than 33 %, while persistent statin users with low-adherence have an adherence of 33 % till 80 %. Statin type, ESD and adherence were measured over time and are time-varying covariates in the time-dependent Cox regression model.

Patients that had at least two prescriptions of blood glucose lowering drugs excl. Insulins (ATC: A10B) (WHOCC, 2020b), with the second prescription not more than six months after the first, were flagged as having diabetes type II from the moment of their first prescription. Drug treatment for diabetes was measured as a time-dependent variable.

### 2.6 Statistical analysis

The cohorts of primary preventive statin users with and without drug treatment for a MACCE were compared using an independent sample Welch’s *t* test for age and a χ2-test for the other discrete variables, to test for statistically significant different relative frequencies between the two subgroups. We constructed Kaplan-Meier curves for the categorical variables: sex, diabetes, statins, dose and adherence. The constructed survival curves were compared using the log-rank test. Because of non-proportional baseline hazards we fitted a weighted time-dependent Cox proportional hazard model (Dunkler et al., 2018, Schemper et al., 2009). We started with univariable proportional hazard model and compared those hazard ratios with the hazard ratios from a multivariable proportional hazard model. The models were fitted from the first statin prescription till the drug-treatment for a MACCE or were censored when participants left or reached the end of the database. Before computing hazard ratios, the correlation between covariates was checked using Cramer’s V (Cramér, 2016). When there was high correlation between variables, one of them was taken out in the multivariable analysis. We split up the data in to a test and training set, with a ratio of 30–70 %. The Cox proportional hazard models were fitted on the training data. Test data was used for validation and calculating the time-dependent Area Under Curve (AUC) scores. The AUC scores are calculated using a method with Cumulative sensitivity and Dynamic specificity, described as method CD4 by Kamarudin et al. (2017). This AUC score is a measure of performance and shows discriminating power of the Cox model. The confidence intervals around the AUC scores are calculated using the method that is described by Hanley and McNeil (1982). All statistical analysis was done in R version 4.0.4. (R Team, 2006). The code is available on request from the first author.

## 3 Results

After applying in- and exclusion criteria, we selected 39,487 statin initiators who started statin therapy anywhere between 1996 and 2019. From the 39,487 statin initiators, 9,224 (23.4 %) received drug treatment for a MACCE with a mean time to event of 5.1 (Standard Deviation [SD]: 4.2, median: 4.1) years after the index date. The other 30,263 patients did not receive any drug treatment for a MACCE and were censored with a mean follow-up time of 7.1 (SD: 4.6, median: 6.1) years. The included statin initiators have a mean age of 59.2 (SD: 11.1) years and 47.3 % of them were men. In Table 2, we have summarized the distribution of all other characteristics according to the outcome. We found statistically significant differences between the group with and without drug treatment for a MACCE for all included variables. The percentage males and prevalence of diabetes was higher in the group treated for a major adverse cardio-cerebrovascular event, and patients with outcome were older on average. The percentage of patients being non-adherent at the start of their statin therapy is statistically significant, albeit small, higher in the group that received drug treatment for a MACCE (29.1 % vs 27.6 %).

**Table 2:** Baseline characteristics of statin initiators with and without a drug-treated major adverse cardio-cerebrovascular event (MACCE).

	No drug-treated MACCE (N = 30,263)	Drug-treated MACCE (N = 9,224)	p-Value
Age, mean (SD), years	58.6 (11.2)	61.1 (10.8)	<0.001
Sex, Man	14,123 (46.7%)	4,575 (49.6%)	<0.001
Diabetes	7,715 (25.5%)	2,955 (32%)	<0.001
Statins			<0.001
Simvastatin	26,756 (88.4%)	7,164 (77.7%)	
Atorvastatin	2,106 (7%)	1208 (13.1%)	
Rosuvastatin	361 (1.2%)	69 (0.7%)	
Other statin	1,040 (3.4%)	783 (8.5%)	
ESD^†^			<0.001
Low	1,597 (5.3%)	820 (8.9%)	
Medium	28,666 (94.7%)	8,404 (91.1%)	
Adherence			0.005
Low (0–80%)	8,342 (27.5%)	2,680 (29.1%)	
Follow-up time, mean (SD), years	7.1 (4.6)	5.1 (4.2)	
Follow-up time, median, years	6.1	4.1	

**Notes:** †See Table 1 for grouping of doses.

**Abbreviations:** SD, Standard Deviation; MACCE, Major Adverse Cardio-Cerebrovascular Event.

**Fig. 1:**
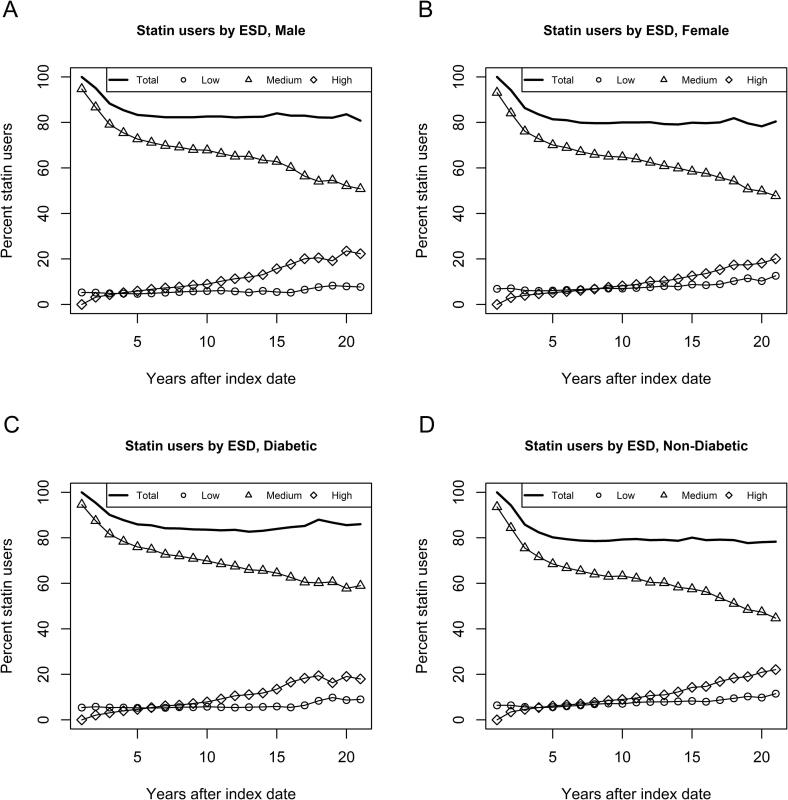
Percentage of primary preventive statin users by statin dose (ESD, Table 1). Figures are shown for males (A), females (B), diabetic (C) and non-diabetic (D) patients.

**Fig. 2:**
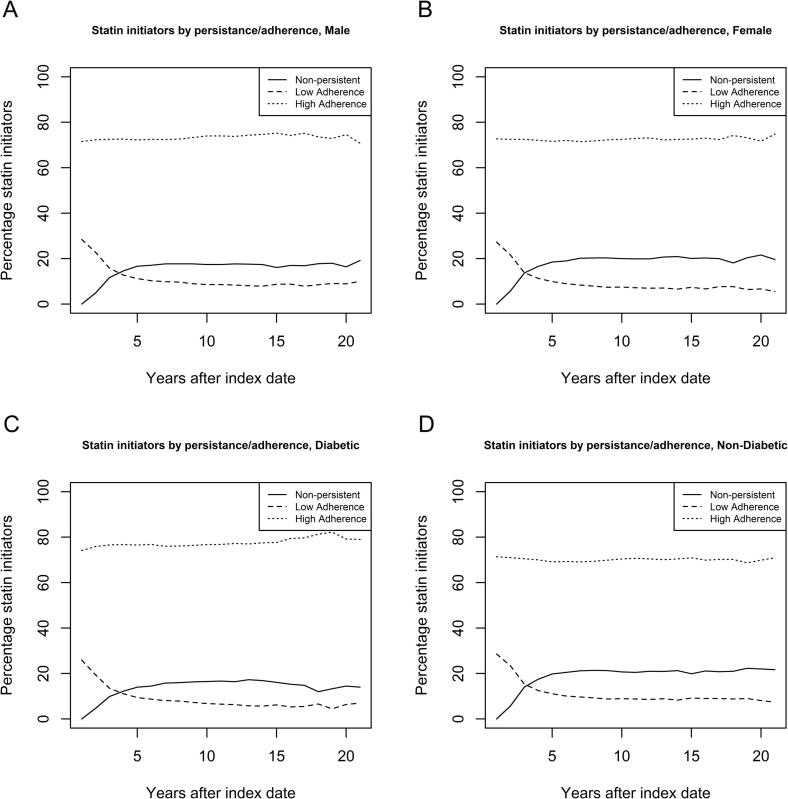
Percentage of statin users with high and low adherence and if they are persistent during follow-up time. Figures are shown for males (A), females (B), diabetic (C) and non-diabetic (D) patients.

Statin usage decreased from 100 % until approximately 81 % after five years. After that, the percentage of statin users remained stable at around 80%. We observed that diabetes patients are a bit more (about 5% on average) persistent than non-diabetic patients (Fig. 1C, D). Furthermore, we observed a decreasing usage of medium ESD statins, while the usage of high ESD statins increases over time for all shown subgroups (Fig. 1). High adherence among the group of statin initiators is constant around 73%. Adherence is higher among diabetes patients than among non-diabetic patients (see Fig. 2C, D). In all subgroups, we observed a decrease in statin initiators with low adherence, while the non-persistency increased (see Fig. 2).

Both the univariable and the multivariable hazard ratios are summarized in Table 3. Because of high correlation between the variables ESD and statin type, we decided to take only the ESD into account when calculating multivariable hazard ratios. Because of the low prevalence in the High ESD class, we combined the classes Medium and High for ESD in the Cox proportional hazard model. Men have a higher risk of receiving drug treatment for a MACCE compared to women (Hazard Ratio [HR]: 1.27, 95 % Confidence Interval [95 %CI]: 1.12–1.44). Also, increasing age was statistically significant associated with a higher risk (HR: 1.03, 95 %CI: 1.02–1.04). The presence of diabetes was associated with an increased risk of receiving drug treatment for a MACCE (HR: 1.38, 95 %CI: 1.24–1.56). There was no statistically significant difference in risk of drug treatment for major adverse cardio-cerebrovascular events between the high and low adherence class. Persistence on the other hand was shown to decrease the risk of receiving drug treatment for a MACCE, with point estimates of the hazard ratio of 0.70 (95 %CI: 0.51–0.95) and 0.71 (95 %CI: 0.58–0.88) for the low, respectively, the medium/high ESD. The three predominantly used statins, simvastatin, atorvastatin and rosuvastatin, all have a significant effect on reducing incident drug treatment for major adverse cardiac and cerebrovascular events, compared to non-persistent patients (see Table 3).

**Table 3:** Unadjusted and adjusted weighted hazard ratios of drug-treated major adverse cardio-cerebrovascular events when on primary preventive statin therapy.

	Weighted Univariable		Weighted multivariable^†^	
	HR (95%-CI)	p-Value	HR (95%-CI)	p-Value
Age	1.03 (1.02–1.04)	<0.001	1.03 (1.02–1.04)	<0.001
Men	1.14 (0.99–1.31)	0.05	1.27 (1.12–1.44)	<0.001
Diabetes	1.43 (1.28–1.63)	<0.001	1.39 (1.24–1.56)	<0.001

No Statin/Non-persistent	1^‡^			N/A
Simvastatin	0.67 (0.58–0.78)	<0.001	N/A	
Atorvastin	0.76 (0.61–0.96)	0.019	N/A	
Rosuvastatin	0.66 (0.49–0.89)	<0.001	N/A	
Other statin	0.81 (0.67–1.00)	0.05	N/A	

No Statin/Non-persistent	1^‡^		1^‡^	
Low ESD	0.72 (0.56–0.92)	<0.001	0.70 (0.51–0.95)	0.023
Medium/High ESD	0.71 (0.61–0.82)	<0.001	0.71 (0.58–0.88)	0.001

No Statin/Non-persistent	1^‡^		N/A	
Low Adherence (0–80%)	0.66 (0.54–0.81)	<0.001	1^‡^	
High Adherence (80%+)	0.71 (0.62–0.82)	<0.001	0.99 (0.83–1.18)	0.891

**Notes:** † Mean time-dependent AUC score on the test set was 0.611 (95% CI: 0.599–0.622), similar to the mean time-dependent AUC score on the training set (0.611, 95% CI: 0.604–0.619).

‡ Reference category.

**Abbreviations**: HR: Hazard Ratio; 95% CI: 95% Confidence Interval; N/A: Not Applicable; AUC: Area Under Curve.

## 4 Discussion

Using this real-world population prescription database, almost one in four patients received drug treatment for major adverse cardio-cerebrovascular events with a median of 4.1 (mean: 5.1) years after starting primary preventive statin treatment. Traditional known risk factors for cardiovascular events in non-statin users such as age, diabetes and sex (Nederlandse Huisartsen Genootschap, 2019, Visseren et al., 2021) remain significantly associated with a higher risk of cardiovascular events within the primary preventive statin group. Patients with diabetes type 2 had a 1.3 times higher risk for cardio-cerebrovascular events which is a lower risk than found in statin untreated populations (between 1.50 and 6.59) (Dugani et al., 2021, Nakazato et al., 2014, Wilson et al., 1998). Both age and sex had similar point estimates of the HR as found in untreated high-cholesterol patients as found by Wilson et al., 1998, Nakazato et al., 2014 and the HR for non-fatal IHD in primary preventive statin patients by Corrao et al. (2010).

The three predominantly used statins in the Netherlands, simvastatin, atorvastatin and rosuvastatin (GIPdatabank, 2021), all have a significant effect on reducing incident drug treatment for major adverse cardio-cerebrovascular events, compared to non-persistent patients (HR: 0.67, 0.76 and 0.66 respectively). This is in line with the results of multiple studies (Aguilar-Palacio et al., 2020, Penning-van Beest et al., 2007, Perreault et al., 2008) and a *meta*-analysis (Martin-Ruiz et al., 2018) that indicate that persistent statin use reduces the risk of e.g. cardiovascular events, hospital admission for acute myocardial infarction and chronic heart failure.

We were unable to detect a difference between patients with a low adherence and those with high statin adherence in terms of risk of drug treatment for a MACCE. The results are similar as those found by Corrao et al. (2010) for the risk of non-fatal IHD, when comparing their four adherence classes to our three classes of non-persistent, low adherence and high adherence and are approximately the same as the results found by Rannanheimo et al. (2015) for the risk of any MACCE or death.

The non-significant difference in relative risk of drug treatment for a MACCE between patients with a low ESD and a medium/high ESD was rather surprising. To the best of our knowledge, no research has been done on the effect of statin dose in primary preventive patients. Nevertheless, the *meta*-analyses of Ribeiro et al., 2013, de Vries et al., 2014 in mainly secondary preventive statin users showed that an increasing dose decreases the relative risk of cardiovascular events.

With a mostly ageing Western population and an increasing risk of cardiovascular events despite statin treatment, older patients should be closely watched. Orkaby et al. (2020) described the potential benefits of statins in a population of age 75 and older, which showed on average a risk reduction of 8 % within six years compared to non-treatment.

We observed a decrease of persistent patients over time with a steady state of about 81 % after five years. In a study by Alfian et al. (2018), which is done using the same IADB.nl database, the authors found a non-persistence rate of about 30 %, three years after statin initiation. However, the investigators only examined a patient population with diabetes type 2, included patients both on primary and secondary statin treatment and reviewed data until 2014. Consequently, we found a non-persistence rate which was lower (12.2 %) after three years in the subgroup of patients with diabetes type 2 within our dataset of primary preventive statin users.

Finally, it seems that patients that are non-adherent at the start of their treatment are more likely to become non-persistent. Since the percentage of high adherent statin users is constant during the time patients are being followed. While the percentage of non-adherent patients decreases with the same rate as the percentage of non-persistent patients increases.

### 4.1 Strengths and limitations

A potential strength of the current study is that it is conducted using real-world data and patients are dispensed statins by a community pharmacist. Findings are therefore applicable to populations with the same characteristics and statin prescription policy (Cohen et al., 2015). Furthermore, we used a weighted cox regression model instead of the standard model to account for non-proportional hazards (Schemper et al., 2009) in combination with time dependent variables for diabetes type 2 drug treatment, the statin dosage and adherence. Since we included almost 40,000 patients into the cohort and one in four developed an outcome, the precision of the estimates were high.

Using drug treatment for major adverse cardio- cerebrovascular events as our primary outcome gave us the opportunity to use more accurate time-dependent data in relation to the outcome events. However, not all patients who received this kind of treatment will have had myocardial infarction or stroke, and some may have ended up in the hospital or died without consultation by a GP and/or pharmacist and consequently will have been missed, as shown by an earlier validation study (Pouwels et al., 2016). Hence, our estimate of one in four drug-treated major adverse cardio-cerebrovascular events in 4 years may translate into higher rates of actual cardio– and cerebrovascular events.

Almost one in four patients in our study received drug treatment for a major adverse cardio-cerebrovascular event. This is almost ten times higher than the clinical cardio- and cerebrovascular event rate found in a *meta*-analysis of randomized controlled trials (Taylor et al., 2013). Explanations could be that the trial populations are not fully representative for the real-world population and therefore differ from our “real world” population. This can be a result of the fact that our “real world” population are only prescribed statins when they are already on a high risk of cardiovascular events, according to the Dutch College of GP guidelines (Nederlandse Huisartsen Genootschap, 2019). Also, trials have stricter treatment protocols, where adherence is optimized most of the time. Compared to the real-world data analysis by Orkaby et al. (2020), which observed an event rate of 42 % for myocardial infarction, ischemic strokes and revascularization procedures combined with a mean follow-up time of nearly seven years in a population older than 75 years, our results could be plausible.

## 5 Conclusion

After statin therapy initiation, in one in four patients incident drug treatment for a major adverse cardio-cerebrovascular event occurred with a median of four years. To reduce event rates in this group, older patients, males and diabetes patients should be closely monitored. Non-adherence in the early stage of treatment should be avoided to prevent non-persistence.

## CRediT authorship contribution statement

**Dennis Steenhuis:** Conceptualization, Methodology, Formal analysis, Writing – original draft, Visualization. **Stijn de Vos:** Conceptualization, Methodology, Validation, Formal analysis, Writing – review & editing, Supervision. **Jens H.J. Bos:** Resources, Data curation. **Eelko Hak:** Conceptualization, Methodology, Validation, Writing – review & editing, Supervision.

## Declaration of Competing Interest

The authors declare that they have no known competing financial interests or personal relationships that could have appeared to influence the work reported in this paper.

## Data Availability

Data will be made available on request.
